# Stable Isotope Provenance of Unidentified Deceased Migrants—A Pilot Study

**DOI:** 10.3390/biology12111371

**Published:** 2023-10-26

**Authors:** Zuzana Obertová, Grzegorz Skrzypek, Martin Danišík, Kai Rankenburg, Marco Cummaudo, Lara Olivieri, Debora Mazzarelli, Annalisa Cappella, Noreen Evans, Douglas Ubelaker, Cristina Cattaneo

**Affiliations:** 1 Department of Biomedical and Health Sciences, University of Milan, 20133 Milan, Italy; 2 Centre for Forensic Anthropology, School of Social Sciences, The University of Western Australia, Crawley 6009, Australia; 3 West Australian Biogeochemistry Centre, School of Biological Sciences, The University of Western Australia, Crawley 6009, Australia; 4 School of Earth and Planetary Sciences, Curtin University, Bentley 6102, Australia; 5 Anthropology, Smithsonian Institution, National Museum of Natural History, Washington, DC 20560, USA

**Keywords:** provenance, human remains, isotopes, Africa, migration

## Abstract

**Simple Summary:**

In the global migration crisis, one of the challenges in the effort to identify deceased migrants is establishing their region of origin, which facilitates the search for ante-mortem data to be compared with the post-mortem information. This pilot study explores the potential of using stable isotope analysis to distinguish individuals coming from West Africa and the Horn of Africa. Six individuals (four of known and two of unknown origin) were sampled for the analysis of stable nitrogen, carbon, oxygen, and strontium isotopes in hair, bone, and dental enamel. The results of the study showed that the stable isotope compositions of the individual from the Horn of Africa differed from the other individuals. The differences found between strontium isotopic composition in enamel and bone, as well as the variations in stable oxygen and carbon isotopes in bone and hair, reflect changes in sources of food and water, in accordance with regionally typical migration journeys. The analysis of multiple stable isotopes delivered promising results, allowing to narrow down the region of origin of deceased migrants and to corroborate the information about the migration journey.

**Abstract:**

In the global migration crisis, one of the challenges in the effort to identify deceased migrants is establishing their region of origin, which facilitates the search for ante mortem data to be compared with the post mortem information. This pilot study explores the potential of using stable isotope analysis to distinguish between individuals coming from West Africa and the Horn of Africa. Six individuals (four of known origin and two of unknown origin) were sampled. δ^13^C_VPDB(keratin)_, δ^15^N_VPDB(keratin)_ and δ^18^O_VSMOW(keratin)_ of hair were analysed using Elemental Analyzers coupled with Isotope Ratio Mass Spectrometry (IRMS). δ^18^O_VSMOW(carbonate)_ and δ^13^C_VPDB(carbonate)_ of bone were analysed using GasBench II with IRMS, while ^87^Sr/^86^Sr composition was determined in bone and dental enamel using laser ablation multi-collector inductively coupled plasma mass spectrometry. The stable isotope compositions of the individual from the Horn of Africa differed from the other individuals. The differences found between ^87^Sr/^86^Sr of enamel and bone and between δ^18^O and δ^13^C in bone and hair reflect changes in sources of food and water in accordance with regionally typical migration journeys. The analysis of multiple stable isotopes delivered promising results, allowing us to narrow down the region of origin of deceased migrants and corroborate the information about the migration journey.

## 1. Introduction

More than 17,000 migrants were reported dead or missing while attempting to cross the Mediterranean Sea from Africa to Europe between 2014 and 2018. The deadliest stretch of the sea was the Central Mediterranean migration route [1,2]. In 2015, the Central Mediterranean route was used mainly by Eritreans, Nigerians and other migrants from East and West Africa. Of the almost 4000 migrants who died while crossing the Mediterranean Sea in 2015, 32% came from Western, Central or Southern Africa, 10% from Eritrea, and 35% were of unknown origin [3]. Of those migrants who reached Italy in 2016, the majority came from Nigeria (21%), Eritrea (11%), Gambia, Cote d’Ivoire, Guinea (7% each), Senegal, and Mali (6% each) [4].

On average, more than 55% of migrants registered as deceased in European countries remain unidentified [3] mainly due to the lack of resources to perform thorough autopsies and to create databases of the existing postmortem findings, and due to the complex and complicated acquisition of comparative ante mortem information. One of the major challenges in the identification process is to establish the region of origin of the individual deceased migrants. Information about geographic origin would facilitate a targeted search for ante mortem data and, subsequently, the comparisons with post mortem findings. It would be of benefit if at least the two predominant regions of migrants’ origin—West Africa and the Horn of Africa—could be differentiated on an individual basis.

A number of publications have demonstrated the value of stable isotope analysis in assessing the provenance of unidentified human remains in forensic cases [5,6,7,8,9]. In particular, the multi-tissue multi-isotope approach has been advocated as different tissues (hair, dental enamel, bone) can provide information about different phases of an individual’s life history, and, since the individual isotopes behave independently of each other, they can be seen as multiple sources of evidence in reconstructing the dietary habits and geographic movements of an individual [9,10,11,12,13]. Notably, the results of stable isotope analyses help narrow down a geographic area of interest (often by excluding particular regions) rather than identify a specific location [12,14,15,16].

Stable isotope analysis of human dental enamel provides information about the dietary and environmental conditions during the mineralization of the enamel, which, depending on the tooth, can correspond to the gestation period up to adolescence and remains constant after the mineralization is completed [12,17]. In contrast, the isotopic analysis of human bone reflects a longer period of time (up to 20 years, depending on the age, type of bone, and turnover rate), and as bone remodels, the signal may change according to the prevailing living conditions [18]. The stable isotope composition of hair provides information about the more recent personal history, ranging from a few days to several months (depending on the sampling strategy and the length of hair). The isotopic values are derived from keratin, which is not easily degraded and is not metabolically active [19,20,21]. 

Stable carbon and nitrogen isotopic values can be considered indirect indicators of geographic origin in cases where a change in diet is observed and is less likely attributed to a simple change in personal dietary preferences [16,22]. The δ^15^N values reflect the proteins in diet, while δ^13^C values based on the analysis of carbonates reflect all dietary components including proteins, carbohydrates, and lipids as opposed to δ^13^C values in collagen, which reflect the proteins only [23,24,25]. 

Stable carbon isotopes are mainly interpreted as differentiating between a diet consisting of C3 or C4 plants (which follow different photosynthetic pathways), or, to a lesser degree, between a diet of marine fish and seafood as opposed to a diet consisting mainly of terrestrial animal products [26,27]. In comparison, δ^15^N values are used as an indicator of the trophic level and the main source of information regarding the proportion of marine versus terrestrial diet. A diet consisting mainly of freshwater fish will return similar δ^13^C and δ^15^N values as a diet based on terrestrial animals [28,29]. Apart from dietary habits, δ^15^N and δ^13^C values can also reflect environmental (e.g., soil fertilization practice) or metabolic factors, such as nutritional stress [30,31].

Stable oxygen isotopes, which reflect ingested water, are frequently used for provenance studies by comparing the values derived from human tissues to global or local precipitation models [32,33,34]. Environmental δ^18^O values vary depending on the continentality (distance from the ocean), altitude, latitude, and season [35,36,37]. 

Strontium isotope (^87^Sr/^86^Sr) values are used as reliable markers of geographic origin, since they are thought to be constant across trophic levels and thus correlate well with the geological signal, which depends on the geochemical composition and age of the bedrock [17,38]. Strontium isotopes enter the human body through the food chain as other isotopes, but the process starts from rock weathering, which causes the release of strontium into soil, and through soil it enters human food sources, including plants, animals, and water [17,39,40]. Individuals who show a difference in ^87^Sr/^86^Sr values between dental enamel and bone bioapatite are assumed to have moved from their original location [17,41]. 

The aim of this pilot study is to explore whether the analysis of δ^18^O, δ^13^C, δ^15^N, and ^87^Sr/^86^Sr in hair, bone and dental enamel would allow inferences regarding the region of origin of deceased migrants and their migration journey. In particular, the potential of the methodology to distinguish individuals coming from two African regions—West Africa and the Horn of Africa—was assessed to facilitate the search for antemortem information necessary for successful identification. 

## 2. Materials and Methods

### 2.1. Materials

Scalp hair, a cross-section of a long bone and a tooth were sampled from six individuals from the forensic cases or collection (CAL) of LABANOF (Laboratorio Antropologia and Odontologia Forense) of the University of Milan, Italy. Four were unclaimed identified individuals, with information on the country of origin obtained from identification documents (one from Eritrea, one from Côte d’Ivoire, and two from Mali). Two individuals were unidentified. For all open cases and individuals recovered from the Mediterranean Sea, the presented isotope analysis was functional to the biological profile; hence, it was authorised by the judicial authority for identification purposes as are all other scientific identification attempts at identification of these victims. The sample characteristics are summarized in Table 1. 

Premolars were sampled because they mineralize during early childhood between two and seven years of age [42], and, thus, most likely do not include the period of breastfeeding.

### 2.2. Sample Preparation

For each individual, approximately 20 scalp hairs (1.2 mg) were sampled, when possible with the root, and were homogeneously trimmed to approximately 3 cm from the root. It is acknowledged that since about 11% of human hair can be in the telogen phase (with 88% in the anagen phase) at any given time, the proportion of hair in telogen will cause an admixture of isotopic signal that will lag behind about 0 to 3 months in reflecting the prevalent diet [22]. The sample was then divided into two vials, one used for δ^15^N and δ^13^C, and one for δ^18^O analysis. A complete premolar (permanent maxillary or mandibular first or second) from each individual was embedded in epoxy resin for the ^87^Sr/^86^Sr analysis.

One approximately 3 mm thick cross-section from the middle part of the diaphysis of dry long bones (femur or tibia) was cut with a laboratory saw. The cross-sections were divided into two halves. One-half was embedded in epoxy resin for the ^87^Sr/^86^Sr analysis. From the second half, the middle part of the cortical bone (the outer layer and inner layer were avoided) was crushed into 1–5 mm pieces of bone tissue (~0.5 mg in total), which were directly caught into a clean labelled vial. 

Prior to the stable isotope analyses, the crushed bone samples were treated with 10% hydrogen peroxide to remove organic compounds, washed with deionized water, dried, and powdered. The hair samples were soaked in a chloroform:methanol mixture to remove contaminations and lipids, washed, dried, and cut into 2 mm pieces [43]. 

### 2.3. Stable Carbon, Nitrogen, and Oxygen Isotope Analyses 

Stable carbon, nitrogen, and oxygen isotope compositions of hair and bone samples were analyzed in the West Australian Biogeochemistry Centre at The University of Western Australia using three different Isotope Ratio Mass Spectrometry (IRMS) systems. All stable isotope compositions were reported using standard δ-notation after multi-point normalization of raw isotope data to one of the international stable isotope scales [44].

Hair samples for δ^15^N and δ^13^C were analyzed using an IRMS Delta V Plus connected via Conflo IV to an elemental analyzer (EA) Thermo Flush 1112 (Thermo-Fisher Scientific, Bremen, Germany). The gases yielded in the EA during sample combustion (1000 °C) were carried in a stream of helium (grade 99.999% purity; BOC, Perth, Australia, 100 mL/min) through reduction columns (650 °C), water traps and then N_2_ was separated from CO_2_ on the GC column before introduction to IRMS as transient peaks [45]. Raw data were obtained using the instrumental software (Isodat 2.5, Thermo Scientific, Bremen, Germany) “SSH” ^17^O correction for CO_2_. Reference materials (δ^13^C—NBS22 −30.03‰, USGS24 −16.05‰, NBS19 1.95‰, LSVEC −46.60‰; δ^15^N—N1 0.43‰, N2 20.41‰, USGS32 180.00‰) provided by the International Atomic Energy Agency (IAEA) were analyzed twice each and used for normalization to international scales defined versus atmospheric air for nitrogen and VPDB for carbon [46,47]. The analytical uncertainty was ≤0.10‰ (1σ).

Hair samples for δ^18^O analysis and three replicates of two international hair standards (USGS42 and USGS43) were weighed into open silver capsules, left for equilibration with air moisture in a sealed plastic box, and then freeze-dried (modified after [48]. The stable oxygen isotope composition of hair samples and standards was analyzed using Thermal Conversion Elemental Analyzer (TC/EA) coupled with IRMS Delta XL (Thermo-Fisher Scientific, Germany). The hair material was converted at 1350 °C to CO gas and after separation, CO was introduced to IRMS. Two reference materials, IAEA601 (23.14‰), IAEA602 (71.28‰) from IAEA were used for normalization to the VSMOW scale [49,50] and then USGS42 (8.56‰) and USGS43 (14.11‰) standards were used for correction of δ^18^O values for exchangeable–OH groups [48]. The analytical uncertainty was ~0.40‰ (1σ).

Carbonates from bone were analyzed for δ^13^C and δ^18^O using GasBench II coupled with IRMS Delta XL (Thermo-Fisher Scientific, Germany). Samples of carbonate powder were flushed with ultra-high purity helium in 12 mL vials and reacted with 100% orthophosphoric acid at 50 °C for 1 h [45,51]. Then, the yield purified CO_2_ gas was analyzed on IRMS. International reference materials from IAEA NBS18 (−5.01‰), NBS19 (1.95‰), and L-SVEC (−46.60‰) for δ^13^C, and NBS18 (−23.2‰), NBS19 (−2.20‰) for δ^18^O were each analysed twice [44] and used for normalization to the VPDB scale. The analytical uncertainty was ≤0.10‰ (1σ).

In bioapatite, δ^18^O values can be measured either in carbonates or in phosphates. The analysis of carbonates is technically simpler, faster and less expensive, and it also allows for simultaneous measuring of δ^13^C values. The analysis of phosphates is recommended for poorly preserved, likely diagenetically altered bone samples from archaeological contexts rather than in modern forensic cases [33,52].

### 2.4. Stable Strontium Isotope Analysis

Complete premolars and bone sections were embedded in epoxy resin (Araldite^®^ DBF, Huntsman Advanced Materials, Modena, Italy) and cured at room temperature. The resin blocks were ground and polished using a Struers DAP-7 grinding wheel for geologists with Buehler^®^ (Leinfelden-Echterdingen, Germany) abrasive paper graded 180, 320, 600, 1200, 2400 and 4000 until exposing the surface of samples.

All strontium isotopic data were obtained in dry-plasma mode following established procedures [53] using a NU Plasma II HR multicollector inductively coupled plasma mass spectrometer (MC-ICP-MS) coupled to a RESOlution M-50 excimer (193 nm) laser-ablation system at the GeoHistory Facility, John de Laeter Centre, Curtin University, Perth, Australia. An array of 13 Faraday cups in half-mass spacing was used to simultaneously collect the mass range from 82 to 88. All data were acquired in low resolution as time resolved analyses using integration times of 2 s and a ‘squid’ signal smoothing device. Laser conditions were as follows: spot size 128 microns, fluence 2 J/cm^2^, laser repetition rate 4 to 10 Hz, and path ablation at 4 microns/sec. The laser repetition rate was adjusted to yield comparable ^88^Sr intensities of 4 to 8 V for both standards and samples. Isobaric interferences on Sr isotopes from Kr, REE^2+^, ArCa, Ca dimers, and Rb were subtracted in this order using the Iolite laser ablation data reduction software [54]. All tooth and bone samples were then normalised to a modern shark in-house reference sample with a known enameloid ^87^Sr/^86^Sr of 0.709174 ± 9 (2 s; n = 3; normalized to SRM987 = 0.710248). Profiles of ^87^Sr/^86^Sr reported here represent moving averages of 5 analyses with a duration of 2 s each, or sections 40 microns in length.

The analytical protocol yielded an excellent external reproducibility of ^87^Sr/^86^Sr of <0.1‰, and an accuracy of 0.0564 for the ^84^Sr/^86^Sr of the shark tooth. Analyses of human teeth and bone can be complicated by low concentrations of Sr, and high concentrations of REE^2+^, Rb, and organic materials. Whereas interference from REE^2+^ could not be detected in our sample runs, Rb levels were elevated (^87^Rb/^86^Sr < 0.012) compared to the shark tooth (^87^Rb/^86^Sr < 0.0001). We therefore determined an adjusted ^87^Rb/^85^Rb fractionation factor from analyses of Rb-spiked SRM987 standard solutions [55] using an Aridus II desolvating nebulizer system (Teledyne CETAC Technologies, Omaha, NE, USA) prior to laser ablation analysis. 

Horstwood et al. [53] reported on the possible occurrence of a Ca-P-O interference at mass 87 leading to correlated offsets in ^87^Sr/^86^Sr with Sr/Ca. In order to account for variable Sr/Ca in standards and samples, igneous apatites (Mud Tank, Otter Lake, AP1) were concomitantly analysed, spanning a much wider range of Sr/Ca and REE contents as those encountered in our sample materials. The results were within 0.4‰ (2σ) of certified ^87^Sr/^86^Sr ratios [56]. Consequently, a conservative error estimate of 0.4‰ (2σ) was assigned to the absolute ^87^Sr/^86^Sr in human bone and teeth samples. The within-run precision of any given bone or tooth profile, however, is assumed to be similar to internal counting statistics of standard runs (<0.14‰).

### 2.5. Data Conversion

The metabolic processes of the formation of different types of human tissues from substrates acquired from food and water are associated with stable isotope fractionations, leading to differences between food sources and tissues and also between different types of tissues [39,52,57]. Therefore, to compare the stable isotope compositions of different tissues within an individual, values need to be converted to address these stable isotope fractionations. The δ^13^Cvalue in bone carbonate can be compared with the δ^13^C value of hair keratin after converting δ^13^C_VPDB(carbonate)_ to δ^13^C_VPDB(keratin)_. In this study, the conversion was conducted as per the equation [Equation (1)] proposed by Lee-Thorp et al. [24] and O’Connell and Hedges [19].

δ^13^C_VPDB(keratin)_ = [(δ^13^C_VPDB(carbonate)_ − 6.4)/1.07] − 1.4). (1)

The stable oxygen composition of hair keratin and bone can be compared after recalculating the original drinking water composition available to a human during respective times of their formation. The δ^18^O values for hair keratin are expressed on the VSMOW scale, while the δ^18^O values for bone carbonate are expressed on the VPDB scale. In the first step, the δ^18^O values of bone carbonate were converted from the VPDB scale to the VSMOW scale (Equation (2)) [36]. In the second step, the δ^18^O values in hair keratin (Equation (3)) and bone carbonate (Equation (4)) were further converted to δ^18^O values of drinking water (DW) following Ehleringer et al. [21], and Chenery et al. [52], respectively.

δ^18^O_VSMOW_ = 1.03086 × δ^18^O_VPDB_ + 30.86 (2)

δ^18^O_VSMOW(DW-keratin)_ = (δ^18^O_VSMOW(keratin)_ − 15.2)/0.353 (3)

δ^18^O_VSMOW(DW-carbonate)_ = 1.590 × δ^18^O_VSMOW(carbonate)_ − 48.634 (4)

## 3. Results

The δ^13^C, δ^15^N, δ^18^O and ^87^Sr/^86^Sr were analysed in respective tissues (Table 2) and then for direct comparison δ^13^C_VPDB(carbonate)_ was converted to δ^13^C_VPDB(keratin)_ and both δ^18^O_keratin and_ δ^18^O_carbonate_ to δ^18^O_DW_ (Equations (1)–(4)). The relative differences between the initial values acquired from the region of origin were compared with the most recent signal (δ^18^O of dental enamel to δ^18^O of carbonate; δ^13^C and δ^18^O of carbonate to δ^13^C and δ^18^O of keratin).

The δ^15^N_AIR(keratin)_ values ranged from 7.72‰ in HA1 to 9.56‰ in WA3. The δ^13^C_VPDB(keratin)_ values were similar among the individuals, ranging from −23.08‰ in WA3 to −21.05‰ in WA1. The δ^13^C_VPDB(carbonate)_ values of bone converted to keratin values showed greater variability than the δ^13^C values in hair, ranging from −22.55‰ in NN1 to −16.10‰ in NN2. 

The δ^18^O_VSMOW(keratin)_ values converted to δ^18^O_VSMOW(keratin-DW)_ showed a wide variation from −13.14‰ in NN1 to −1.06‰ in HA1, while the δ^18^O_VSMOW(carbonate)_ values converted to δ^18^O_VSMOW(keratin-DW)_ varied from −8.51‰ in NN1 to −2.87‰ in HA1. 

The ^87^Sr/^86^Sr in dental enamel ranged from 0.711 in HA1 to 0.723 in WA2 and WA3. All individuals showed lower values of ^87^Sr/^86^Sr in bone than in teeth; the bone values ranged from 0.710 in HA1 to 0.720 in WA3. 

**Figure 1:**
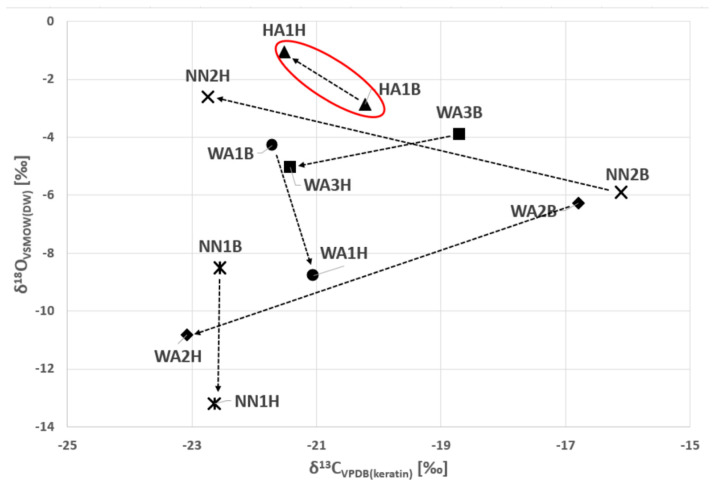
The comparison between the stable carbon and oxygen isotope compositions of hair (H) keratin (temporally more recent composition) and bone (B) carbonates (the ‘region of origin’ composition) for the six individuals. The original values analysed in tissues were converted as follows: δ^13^C_VPDB(carbonate)_ to δ^13^C_VPDB(keratin)_ and δ^18^O_VSMOW(carbonate)_ and δ^18^O_VSMOW(keratin)_ to δ^18^O_VSMOW(DW)_. The arrows show the changes over time between bone and hair signals. Legend: DW—drinking water; HA—Horn of Africa (red oval); WA—West Africa; NN—Unknown.

**Figure 2:**
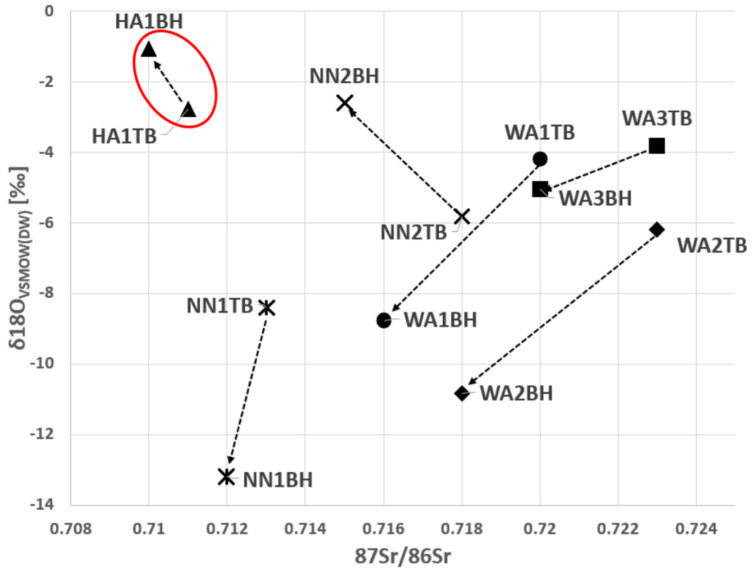
The comparison between the initial (‘region of origin’) composition of ^87^Sr/^86^Sr in dental enamel and δ^18^O_VSMOW(carbonate)_ to the more recent composition of ^87^Sr/^86^Sr in bone and δ^18^O_VSMOW(keratin)_. The δ^18^O_VSMOW(carbonate)_ and δ^18^O_VSMOW(keratin)_ values were converted to δ^18^O_VSMOW(DW)_. The arrows show the changes over time in the isotopic signals. Legend: DW—drinking water; TB—tooth and bone; BH—bone and hair; HA—Horn of Africa (red oval); WA—West Africa; NN—Unknown.

As shown in Figure 1 and Figure 2, the samples from the young adult male from the Horn of Africa (HA1) can be clearly distinguished from the West African males as well as from the unknown males primarily due to the original isotopic signal of δ^13^C and δ^18^O in bone and ^87^Sr/^86^Sr in dental enamel (Figure 1 and Figure 2). In Figure 2, the ^87^Sr/^86^Sr in dental enamel and δ^18^O_VSMOW(carbonate)_ values represent an approximation of the original isotopic signals, while the ^87^Sr/^86^Sr in bone and δ^18^O_VSMOW(keratin)_ represent the more recent signals. Although it is acknowledged that the time frames of isotopic intake do not entirely overlap between enamel and bone and bone and keratin, respectively, the shift in the isotopic signal is clearly discernible.

## 4. Discussion

This pilot study used multi-tissue, multi-isotope analysis to explore its potential to trace the provenance of unidentified deceased migrants, and thus facilitate identification. The differentiation between two geographic regions—the Horn of Africa and West Africa—was targeted. 

The stable isotope values of the individual from the Horn of Africa (HA1) differed from the values of the West African individuals. The tissue samples from this individual were characterised by the lowest ^87^Sr/^86^Sr ratio in dental enamel and bone, and the least negative δ^18^O values in bone carbonate as well as in hair, reflecting a large difference in δ^18^O of mean precipitation between these two regions (δ^18^O > 5‰). In addition, the δ^15^N values in hair keratin for HA1 were also the lowest among all individuals. 

### 4.1. Food Sources

The stable nitrogen and carbon isotope compositions provided information regarding the prevalent diet composition [27,28]. In principle, there are some differences in the traditional cuisine between the Horn of Africa and the West African countries as well as among certain regions within West Africa, including the proportion of C3 and C4 plants in the diet, and the proportion of consumed meat and fish [58,59,60,61]. Differences in prevalent diet may, therefore, provide some indication of the region of origin, but personal and cultural dietary preferences may also lead to local interindividual variability in δ^15^N and δ^13^C signals [16,22]. 

In the Horn of Africa, the diet is primarily based on C3 plants (wheat, barley or rice), but maize and sorghum (i.e., C4 plants) are also commonly consumed [35,59,60]. In general, there are two distinct crop zones in West Africa: rice (C3 plant) is the main crop in the region of Senegambia, through Guinea up to Cote d’Ivoire, while the remaining countries, including Mali and Nigeria belong to the millet/sorghum/maize (C4) crop zone [35]. Palm nut (oil) and starchy tubers like yams, plantains, sweet potatoes, peanuts and cassava, which are typical C3 plants or plants with an intermediate signal between C3 and C4 plants, are staple foods in many West African countries [35,58]. 

Vegetables are common in West African cuisine, especially squash and green leafy vegetables. In the Horn of Africa, fruit is commonly part of the diet, and if meat is not available, it is substituted by legumes. In the Horn of Africa, fish is rarely consumed, while meat is commonly eaten. In contrast, in West Africa, fish is popular in the coastal areas and along the major rivers of the region, while in the inland areas, meat is preferred but not always available [58,59,60,61]. 

The δ^13^C in the bone carbonate of HA1 was more negative than the values of WA2 and WA3 but less negative than that of WA1. This indicates that HA1 consumed more likely C3 rather than C4 plants than WA2 and WA3, which would be in accordance with the prevalent diet in the Horn of Africa. Except for HA1, all other individuals showed δ^15^N signals similar to those observed in European omnivorous and vegetarian humans (8.2–9.4‰ [19]). The δ^15^N_AIR(keratin)_ of 7.72‰ recorded for HA1 is consistent with values below 8‰ found in European vegans [19]. In the Horn of Africa, socioeconomically disadvantaged groups often have a vegan diet, using legumes as a substitution for meat [61]. Since δ^15^N was not measured in bone, we have only information about the most recent isotopic signal. 

### 4.2. Water Sources

On average, the δ^18^O values of drinking water are expected to be more negative in West Africa compared to the Horn of Africa [34,35,62], which was reflected in the more negative values of δ^18^O_VPDB_ in the bone carbonate of the West African individuals compared to HA1. Although recalculating δ^18^O from keratin and carbonate to δ^18^O_VSMOW(DW)_ may introduce some error [63], the recalculation followed robust standards and the results were consistent with the expected values. 

In general, the more inland and the higher the elevation, the more negative the δ^18^O values from precipitation are expected due to continental and rain-out effects [34,37,64,65]. The δ^18^O composition of water from (deep) groundwater sources is usually more negative than the signals of surface water and meteoric water [34,64]. However, local drinking water sources could be highly variable, depending on the primary water source used in the region (surface water versus groundwater) due to the progress of evaporation. Jorgensen and Banoeng-Yakubo [64] reported groundwater δ^18^O values of approximately −3.5 to −2.0‰ on the southwestern coast of Ghana in the Volte River estuary, which, they say, are similar to groundwater values in the southern Voltaian Sedimentary Basin, Accra region, and in other coastal basins at the same latitude in West Africa (e.g., in Nigeria), but are slightly less negative in comparison to groundwater values in the Upper West Region and Upper East Region of Ghana, and in other, more continentally located West African countries, such as Niger, Burkina Faso, Mali, and Senegal. The individual from Côte D’Ivoire had a less negative δ^18^O value of bone than the individuals from Mali, which would be in agreement with the less negative median values of oxygen in the groundwater in this country and the proximity to the coast. The NN individuals had δ^18^O values in bone carbonate similar to or lower than one of the individuals from Mali, which may indicate that they originated from more inland areas and possibly higher altitude areas of West Africa. 

### 4.3. Geological Background

The ^87^Sr/^86^Sr values for countries in West Africa, which mostly lie on the so-called West African Craton, are expected to be comparably higher (bedrock values from 0.7310 to 0.7679) than for countries of the Horn of Africa (bedrock values from 0.7051 to 0.7085, rarely up to 0.7310). However, some areas of Nigeria, Niger, Senegal, and Gambia (bedrock values from 0.7075 to 0.7085) may provide signals similar to the countries of the Horn of Africa [39,66,67]. 

According to IOM [68,69], most migrants from West Africa travel through Niger. The migrants from Senegal, Gambia, Guinea and Côte D’Ivoire usually travel through Mali and Burkina Faso to Niger. The migrants from Eritrea and Somalia travel mainly through Ethiopia and Sudan. The ^87^Sr/^86^Sr values of transit countries vary: the values for parts of Niger and Algeria correspond to the values of the West African Craton, while the values for Libya (bedrock values from 0.7075 to 0.7085), Ethiopia and Sudan are similar to those of the Horn of Africa [39,66]. 

The few ^87^Sr/^86^Sr values reported for water or soil in West African countries are as follows: Pye [70] found a range of values from 0.717 to 0.747 in soil extracts from Nigeria; Goodman et al. [69] reported a value larger than 0.735 for a water sample from Ghana; and Jorgensen and Banoeng-Yakubo [64] showed that ^87^Sr/^86^Sr values in the Volta River Estuary in Ghana varied depending on water source (including infiltration of seawater) and geological substrate, that is shallow groundwater wells, which were usually used for domestic water supply and dry season irrigation returned values between 0.7092 and 0.7098 and 0.7111 and 0.7126, and deep groundwater wells, which were used for urban water supply varied between 0.7079 and 0.7089 and 0.7130 and 0.7140. In addition, Goodman et al. [71] reported ^87^Sr/^86^Sr values for two human teeth from Ghana, with dental enamel values of 0.723 and 0.729, and dentin values of 0.721 and 0.716, respectively. In the present study, the ^87^Sr/^86^Sr values from the three individuals from West Africa varied from 0.720 to 0.723 in dental enamel, while the ^87^Sr/^86^Sr of HA1 was the lowest of all with 0.711, reflecting well the geological composition of the countries of the Horn of Africa.

### 4.4. Provenance of Unknown Individuals

Based on the combination of the observed δ^13^C, δ^18^O and ^87^Sr/^86^Sr values, one of the individuals of unknown origin (NN1) originated from an inland geographic area, potentially of higher elevation, located outside of the West African Craton. The δ^13^C values of NN1 indicate that he most likely consumed a diet based on C3 plants or a mixture of C3 and C4 plants and terrestrial meat sources (with limited or no seafood). The δ^18^O values were the lowest of all tested individuals. As mentioned above, such values are indicative of an inland geographic area, potentially of higher elevation. Ethiopia in the Horn of Africa fits well the description of an inland area with high mountains not located on the West African Craton. Alternatively, NN1 may have come from the inland part of West Africa, most likely Nigeria or Niger and spent long periods of time in an area of high elevation (potentially during his migration journey). 

The ^87^Sr/^86^Sr values of NN1 of 0.713 were similar to those reported by Pye [70] for soil samples from Benin City in southern Nigeria. Although Nigeria/Niger belongs to the C4 crop zone, individual dietary preferences cannot be excluded. Moreover, morphological analysis of the remains of NN1 showed more similarities with other individuals from West Africa than with those originating from the Horn of Africa. 

The isotopic values of the second individual of unknown origin (NN2) corresponded approximately with δ^13^C and δ^18^O values in the bone of WA2, although ^87^Sr/^86^Sr in dental enamel was lower in NN2 than in West African individuals. The ^87^Sr/^86^Sr ratios were, however, higher than would be expected for the countries of the Horn of Africa, so it is likely that the individual came from one of the West African countries, which lie outside of the West African Craton. The δ^18^O values point to an inland area and the δ^13^C values reflect diet based on C4 plants (or fish). 

### 4.5. Temporal Changes in Stable Isotope Compositions

Notably, the δ^13^C and δ^18^O values were measured in samples from the cortical portion of long bones, so they represent a mixture of original and more recent values, which reflect the length of travel and the length of stay in various transit countries of the given individual. Similarly, the shift in ^87^Sr/^86^Sr ratios measured in dental enamel against bone reflects the whole migration journey, rather than differentiating only between the signals of the country of origin versus the last transit country (usually Libya [68,69]). Since the individuals were all young adults and the bone turnover rate in young adults likely spans more than 10 years [18], the isotopic signal of the bones represents a mixture of the developmental signal and the signal acquired during the migration journey, depending on the length of stay in the various transit countries. Consequently, the initial δ^13^C and δ^18^O values of bone represent the isotopic signal acquired during childhood up to the time of death, in comparison to the hair keratin composition representing approximately three months before death [19,20,21,72]. 

The average precipitation δ^18^O values in Libya, the country from which the majority of migrants start their journey across the Central Mediterranean Sea, are similar to those in the West African countries [35,62]. According to a survey of migrants who reached Europe, the average duration of their journey from the country of origin to Libya is about 1.5 years [69]. Migrants from West African countries often spend up to a year in Niger before travelling onward to Libya, while migrants from the Horn of Africa may spend one year or even longer in Sudan or Ethiopia. The West African migrants usually spend a longer time (three to twelve months) in Libya than the migrants from the Horn of Africa [69]. 

Inferring from the δ^13^C values there was a clear change in the diet for two individuals from West Africa and one of unknown origin. The δ^13^C_VPDB (carbonate)_ values for WA2 (−16.79‰), WA3 (−18.71‰) and NN2 (−16.10‰) are typical of a diet based on C4 plants or fish. In comparison, there was little variation in the recent δ^13^C_VPDB (keratin)_ signal among all sampled individuals, ranging from −21.05‰ to −23.08‰. The decrease in δ^13^C values for WA2, WA3 and NN2 can be attributed to a change in diet, specifically to the transition from a diet based on C4 plants (or seafood) to the typical cuisine of Libya, which is based on wheat, barley, and potatoes (i.e., C3 plants). 

The two individuals with Malian identification documents showed some variation in δ^13^C, δ^18^O and ^87^Sr/^86^Sr initial, ‘region of origin’ values; the absolute difference between the individuals being 5.26‰ for δ^13^C_VPDB(carbonate)_, 1.21‰ for δ^18^O_VPDB(carbonate),_ and 0.003 for ^87^Sr/^86^Sr. Mali is a large country, so the two individuals may have originated from different parts of the country. It may be that WA1 potentially originated from the western part of Mali, influenced by the C3 crop zone and possibly fish in diet, lying closer to the coast and rivers (hence, less negative δ^18^O value) and geologically located on the border between the old West African Craton and younger Cenozoic sediments. Another explanation may be that the identification documents were not issued in the country of origin but were acquired during the journey. 

Both individuals from Mali also showed a change in δ^18^O and ^87^Sr/^86^Sr values, indicating a relocation to countries lying more inland (possibly at a higher elevation) and outside of the West African Craton. Approximately one-fifth of the migrants from Mali travel to Libya through Algeria instead of through Niger [69], which would correspond with more negative δ^18^O values and ^87^Sr/^86^Sr strontium values. 

Finally, the more negative δ^18^O values may be explained by physiological rather than spatial reasons. According to the reports by migrants who reached European shores, deprivation of food and water for several days is common during transit [69]. During starvation, renal function deteriorates and as shown for patients with end-stage renal disease, the oxygen isotope values decrease compared to healthy controls [73]. This alternative scenario is also supported by the shift towards more negative δ^13^C values, which occurred to some extent for all tested individuals (except for NN1). Such δ^13^C reduction has been associated with starvation in diseases such as anorexia nervosa [30,74,75]. The δ^18^O values were less negative in hair than in bone in the individual from the Horn of Africa (HA1) and in the individual of unknown origin (NN2) as opposed to the reversed shift in the other four individuals. Less negative δ^18^O values may indicate a longer stay in coastal regions or reflect the fact that these individuals have not experienced episodes of starvation during their journey.

## 5. Conclusions

This multi-tissue multi-isotope analysis has delivered valuable indications concerning the differentiation between geographical regions within Africa. This approach, accompanied by other findings, including anthropological analysis of the remains or personal belongings may help narrow down the search for antemortem information of deceased migrants and corroborate the information about migration journeys. As a pilot study, the sample was small, but the proposed approach of using different tissues and various stable isotopes allowed for several relevant conclusions about the individual samples, which is, and is currently proving to be, key for identification and the understanding of the migration journeys. For a better understanding of the results, more research is needed on the effect of a (prolonged) submersion in seawater on the diagenesis of the examined tissues and, thus, on the interpretation of the measured isotopic signal in this particular sample. Moreover, forensic interpretation would be facilitated by compilation of reference databases of bioavailable stable isotope compositions and isoscapes for specific African regions and countries.

## Figures and Tables

**Table 1:** Sample characteristics—demographic information and tissue sampled.

Individual	Age Group and Sex	Origin	Hair Sample	Tooth Sample	Bone Sample (Chips)	Bone Sample (Section for Sr Analysis)
HA1	young adult male	Eritrea	scalp	left maxillary second premolar	tibia	tibia
WA1	young adult male	Mali	scalp	left mandibular first premolar	femur	femur
WA2	young adult male	Mali	scalp	left mandibular second premolar	femur	femur
WA3	young adult male	Côte D’Ivoire	scalp	left mandibular second premolar	tibia	tibia
NN1	young adult male	unknown	scalp	left mandibular second premolar	femur	femur
NN2	young adult male	unknown	scalp	left mandibular second premolar	femur	femur

**Table 2:** Stable isotope compositions as directly measured in compounds of various human tissues (hair keratin, bone carbonate, and dental enamel).

Individual	Hair Keratin δ^13^C [‰ VPDB]	Hair Keratin δ^15^N [‰ AIR]	Hair Keratin δ^18^O [‰ VSMOW]	Bone Carbonate δ^13^C [‰ VPDB]	Bone Carbonate δ^18^O [‰ VPDB]	Dental Enamel ^87^Sr/^86^Sr	Bone ^87^Sr/^86^Sr
HA1	−21.52	7.72	14.8	−13.74	−2.02	0.711	0.710
WA1	−21.05	8.67	12.1	−15.33	−2.88	0.720	0.716
WA2	−23.08	8.14	11.4	−10.07	−4.09	0.723	0.718
WA3	−21.42	9.56	13.4	−12.12	−2.64	0.723	0.720
NN1	−22.63	8.82	10.5	−16.23	−5.46	0.713	0.712
NN2	−22.75	8.37	14.3	−9.33	−3.87	0.718	0.715

## Data Availability

The data presented in this study are available on request from the corresponding author. The data are not publicly available due to ethical restrictions.

## References

[1] Missing Migrants Project. https://missingmigrants.iom.int/region/mediterranean.

[2] Laczko F., Singleton A., Black J. (2017). Fatal Journeys—Improving Data on Missing Migrants, Volume 3 Part 2.

[3] Brian T., Laczko F. (2016). Fatal Journeys: Identification and Tracing of Dead and Missing Migrants.

[4] UNHCR 2017 Italy Sea Arrivals Dashboard. December 2016. https://data2.unhcr.org/fr/documents/download/53356.

[5] Bartelink E.J., Berg G.E., Beasley M.M., Chesson L.A. (2014). Application of stable isotope forensics for predicting region of origin of human remains from past wars and conflicts. Ann. Anthropol. Pract..

[6] Font L., van den Peijl G., van Leuwen C., van Wetten I., Davies G.R. (2015). Identification of the geographical place of origin of an unidentified individual by multi-isotope analysis. Sci. Justice.

[7] Juarez C.A. (2008). Strontium and geolocation, the pathway to identification for deceased undocumented Mexican border-crossers: A preliminary report. J. Forensic Sci..

[8] Kamenov G.D., Kimmerle E.H., Curtis J.H., Norris D. (2014). Georeferencing a cold case victim with lead, strontium, carbon, and oxygen isotopes. Ann. Anthropol. Pract..

[9] Lehn C., Rossmann A., Graw M. (2015). Provenancing of unidentified corpses by stable isotope techniques-presentation of case studies. Sci. Justice.

[10] Lehn C., Mützel E., Rossmann A. (2011). Multi-element stable isotope analysis of H, C, N and S in hair and nails of contemporary human remains. Int. J. Legal Med..

[11] Laffoon J.E., Sonnemann T.F., Shafie T., Hofman C.L., Brandes U., Davies G.R. (2017). Investigating human geographic origins using dual-isotope (^87^Sr/^86^Sr, δ^18^O) assignment approaches. PLoS ONE.

[12] Schroeder H., O’Connell T.C., Evans J.A., Shuler K.A., Hedges R.E.M. (2009). Trans-Atlantic slavery: Isotopic evidence for forced migration to Barbados. Am. J. Phys. Anthropol..

[13] Lamb A.L., Evans J.E., Buckley R., Appleby J. (2014). Multi-isotope analysis demonstrates significant lifestyle changes in King Richard III. J. Archaeol. Sci..

[14] Meier-Augenstein W., Fraser I. (2008). Forensic isotope analysis leads to identification of a mutilated murder victim. Sci. Justice.

[15] Valenzuela L.O., Chesson L.A., Bowen G.J., Cerling T.E., Ehlinger J.R. (2012). Dietary heterogeneity among Western industrialized countries reflected in the stable isotope ratios of human hair. PLoS ONE.

[16] Hülsemann F., Lehn C., Schneider S., Jackson G., Hill S., Rossmann A., Scheid N., Dunn P.J.H., Flenker U., Schänzer W. (2015). Global spatial distributions of nitrogen and carbon stable isotope ratios of modern human hair. Rapid Commun. Mass Spectrom..

[17] Beard B.L., Johnson C.M. (2000). Strontium isotope composition of skeletal material can determine the birth place and geographic mobility of humans and animals. J. Forensic Sci..

[18] Hedges R.E.M., Clement J.G., Thomas C.D.L., O’Connell T.C. (2007). Collagen turnover in the adult femoral midshaft: Modeled from anthropogenic radiocarbon tracer measurements. Am. J. Phys. Anthropol..

[19] O’Connell T.C., Hedges R.E.M. (2001). Isotopic comparisons of hair, nail and bone: Modern analyses. J. Archaeol. Sci..

[20] Fraser I., Meier-Augenstein W., Kalin R.M. (2006). The role of stable isotopes in human identification: A longitudinal study into the variability of isotopic signal in human hair and nails. Rapid Commun. Mass Spectrom..

[21] Ehleringer J.R., Bowen G.J., Chesson L.A., West A.G., Podlesak D.W., Cerling T.E. (2008). Hydrogen and oxygen isotope ratios in human hair are related to geography. Proc. Natl. Acad. Sci. USA.

[22] O’Connell T.C., Hedges R.E.M. (1999). Investigations into the effect of diet on modern human hair isotopic values. Am. J. Phys. Antropol..

[23] Jim S., Ambrose S.H., Evershed R.P. (2004). Stable carbon isotopic evidence for differences in the dietary origin of bone cholesterol, collagen and apatite: Implications for their use in palaeodietary reconstruction. Geochim. Cosmochim. Acta.

[24] Lee-Thorp J.A., Sealy J.C., van der Merwe N.J. (1989). Stable carbon isotope ratio differences between bone collagen and bone apatite, and their relationship to diet. J. Archaeol. Sci..

[25] Hedges R.E.M. (2003). On bone collagen-apatite-carbonate isotopic relationships. Int. J. Osteoarchaeol..

[26] Schoeninger M.J. (1995). Stable isotope studies in human evolution. Evol. Anthropol..

[27] DeNiro M.J., Epstein S. (1978). Influence of diet on the distribution of carbon isotopes in animals. Geochim. Cosmochim. Acta.

[28] Schoeninger M.J., DeNiro M.J. (1984). Nitrogen and carbon isotopic composition of bone collagen from marine and terrestrial animals. Geochim. Cosmochim. Acta.

[29] Hülsemann F., Koehler K., Braun H., Schänzer W., Flenker U. (2013). Human dietary δ^15^N intake: Representative data for principle food items. Am. J. Phys. Anthropol..

[30] Petzke K.J., Fuller B.T., Metges C.C. (2010). Advances in natural stable isotope ratio analysis of human hair to determine nutritional and metabolic status. Curr. Opin. Clin. Nutr. Metab. Care.

[31] Amundson R., Austin A.T., Schuur E.A.G., Yoo K., Matzek V., Kendall C., Uebersax A., Brenner D., Baisden W.T. (2003). Global patterns of the isotopic composition of soil and plant nitrogen. Global Biogeochem. Cycles.

[32] Kennedy C.D., Bowen G.J., Ehleringer J.R. (2011). Temporal variation of oxygen isotope ratios (δ^18^O) in drinking water: Implications for specifying location of origin with human scalp hair. Forensic Sci. Int..

[33] Lightfoot E., O’Connell T.C. (2016). On the use of biomineral oxygen isotope data to identify human migrants in the archaeological record: Intra-sample variation, statistical methods and geographical considerations. PLoS ONE.

[34] Bowen G.J., Revenaugh J. (2003). Interpolating the isotopic composition of modern meteoric precipitation. Water Resour. Res..

[35] Bowen G.J. (2010). Isoscapes: Spatial Pattern in Isotopic Biogeochemistry. Annu. Rev. Earth Planet. Sci..

[36] International Atomic Energy Agency and United Nations Educational, Scientific and Cultural Organization (2000). Environmental Isotopes in the Hydrological Cycle. Principles and Application.

[37] Gat J.R. (1996). Oxygen and hydrogen isotopes in the hydrological cycle. Annu. Rev. Earth Planet. Sci..

[38] Coelho I., Castanheira I., Bordado J.M., Donard O., Silva J.A.L. (2017). Recent developments and trends in the application of strontium and its isotopes in biological related fields. TrAC Trends Anal. Chem..

[39] Bataille C.P., Bowen G.J. (2012). Mapping ^87^Sr/^86^Sr variations in bedrock and water for large scale provenance studies. Chem. Geol..

[40] Hartman G., Richards M. (2014). Mapping and defining sources of variability in bioavailable strontium isotope ratios in the Eastern Mediterranean. Geochim. Cosmochim. Acta.

[41] Degryse P., De Muynck D., Delporte S., Boyen S., Jadoul L., De Winne J., Ivaneanu T., Vanhaecke F. (2012). Strontium isotopic analysis as an experimental auxiliary technique in forensic identification of human remains. Anal. Methods.

[42] AlQahtani S.J., Hector M.P., Liversidge H.M. (2010). Brief communication: The London atlas of human tooth development and eruption. Am. J. Phys. Anthropol..

[43] Bowen G.J., Chesson L., Nielson K., Cerling T.E., Ehleringer J.E. (2005). Treatment methods for the determination of δ^2^H and δ^18^O of hair keratin by continuous-flow isotope-ratio mass spectrometry. Rapid Commun. Mass Spectrom..

[44] Skrzypek G. (2013). Normalization procedures and reference material selection in stable HCNOS isotope analyses—An overview. Anal. Bioanal. Chem..

[45] Paul D., Skrzypek G. (2007). Assessment of Carbonate-Phosphoric Acid Analytical Technique Performed using GasBench II in Continuous Flow Isotope Ratio Mass Spectrometry. Int. J. Mass Spectrom..

[46] Skrzypek G., Sadler R., Paul D. (2010). Error propagation in normalization of stable isotope data: A Monte Carlo analysis. Rapid Commun. Mass Spectrom..

[47] Coplen T.B., Brand W.A., Gehre M., Groning M., Meijer H.A.J., Toman B., Verkouteren R.M. (2006). New Guidelines for δ^13^C measurements. Anal. Chem..

[48] Coplen T.B., Qi H. (2012). USGS42 and USGS43: Human-hair stable hydrogen and oxygen isotopic reference materials and analytical methods for forensic science and implications for published measurement results. Forensic Sci. Int..

[49] Skrzypek G., Sadler R. (2011). A strategy for selection of reference materials in stable oxygen isotope analyses of solid materials. Rapid Commun. Mass Spectrom..

[50] Brand W.A., Coplen T.B., Aerts-Bijma A.T., Böhlke J.K., Gehre M., Geilmann H., Gröning M., Jansen H.G., Meijer H.A.J., Mroczkowski S.J. (2009). Comprehensive inter-laboratory calibration of reference materials for δ^18^O versus VSMOW using various on-line high-temperature conversion techniques. Rapid Commun. Mass Spectrom..

[51] Paul D., Skrzypek G. (2006). Flushing time and storage effects on the accuracy and precision of carbon and oxygen isotope ratios of sample using the GasBench II technique. Rapid Commun. Mass Spectrom..

[52] Chenery C.A., Pashley V., Lamb A.L., Sloane H.J., Evans J.A. (2012). The oxygen isotope relationship between the phosphate and structural carbonate fractions of human bioapatite. Rapid Commun. Mass Spectrom..

[53] Horstwood M.S.A., Evans J.A., Montgomery J. (2008). Determination of Sr isotopes in calcium phosphates using laser ablation inductively coupled plasma mass spectrometry and their application to archaeological tooth enamel. Geochim. Cosmochim. Acta.

[54] Paton C., Hellstrom J., Paul B., Woodhead J., Hergt J. (2011). Iolite: Freeware for the visualisation and processing of mass spectrometric data. J. Anal. At. Spectrom..

[55] Müller W., Anczkiewicz R. (2016). 2016. Accuracy of laser-ablation (LA)-MC-ICPMS Sr isotope analysis of (bio) apatite–a problem reassessed. J. Anal. At. Spectrom..

[56] Yang Y.H., Wu F.Y., Yang J.H., Chew D.M., Xie L.W., Chu Z.Y., Zhang Y.B., Huang C. (2014). Sr and Nd isotopic compositions of apatite reference materials used in U–Th–Pb geochronology. Chem. Geol..

[57] Daux V., Lécuyer C., Héran M.-A., Amiot R., Simon L., Fourel F., Martineau F., Lynnerup N., Reychler H., Escarguel G. (2008). Oxygen isotope fractionation between human phosphate and water revisited. J. Hum. Evol..

[58] West African Cuisine. https://en.wikipedia.org/wiki/West_African_cuisine.

[59] Somali Cuisine. https://en.wikipedia.org/wiki/Somali_cuisine.

[60] Eritrean Cuisine. http://www.eritrea.be/old/eritrea-cuisine.htm.

[61] Speedy A.W. (2003). Global production and consumption of animal source foods. J. Nutr..

[62] International Atomic Energy Agency (2007). Atlas of Isotope Hydrology—Africa.

[63] Pollard A.M., Pellegrini M., Lee-Thorp J.A. (2011). Technical note: Some observations on the conversion of dental enamel δ^18^O_p_ values to δ^18^O_w_ to determine human mobility. Am. J. Phys. Anthropol..

[64] Jorgensen N.O., Banoeng-Yakubo B.K. (2001). Environmental isotopes (δ^18^O, δ^2^H and ^87^Sr/^86^Sr) as a tool in groundwater investigations in Kota Basin Ghana. Hydrogeol. J..

[65] Gonfiantini R., Roche M.A., Olivny J.C., Fontes J.C., Zuppi G.M. (2001). The altitude effect on the isotopic composition of tropical rains. Chem. Geol..

[66] BRGM (2016). The New 1:10,000,000 Geological Map of Africa. http://www.brgm.eu/project/new-edition-of-110000000-geological-map-of-africa.

[67] Wright J.B., Hastings D.A., Jones W.B., Williams H.R. (1985). Geology and Mineral Resources of West Africa.

[68] International Organization for Migration (2017). Libya 2016: Migration Profiles and Trends. Displacement Tracking Matrix.

[69] Achilli L., Fargues P., Salamonska J., Talo T. (2016). Study on Migrants. Profiles, Drivers of Migration and Migratory Trends.

[70] Pye K., Pye K., Croft D.J. (2004). Isotope and trace element analysis of human teeth and bones for forensic purposes. Forensic Geoscience: Principles, Techniques and Application.

[71] Goodman A., Jones J., Reid J., Mack M., Blakey M.L., Amarasiriwardena D., Burton P., Coleman D., Blakey M.L., Rankin-Hill L.M. (2004). Isotopic and elemental chemistry of teeth: Implications for places of birth, forced migration patterns, nutritional status, and pollution. The New York African Burial Ground Skeletal Biology Final Report.

[72] Stepańczak B., Szostek K., Pawlyta J. (2014). The human bone oxygen isotope ratio changes with aging. Geochronometria.

[73] Kuo T.-C., Wang C.-H., Lin H.-C., Lin Y.-H., Lin M., Lin C.M., Kuo H.-S. (2012). Assessment of Renal Function by the Stable Oxygen and Hydrogen Isotopes in Human Blood Plasma. PLoS ONE.

[74] Hatch K.A., Crawford M.A., Kunz A.W., Thomsen S.R., Eggett D.L., Nelson S.T., Roeder B.L. (2006). An objective means of diagnosing anorexia nervosa and bulimia nervosa using ^15^N/^14^N and ^13^C/^12^C ratios in hair. Rapid Commun. Mass Spectrom..

[75] Mekota A.M., Grupe G., Ufer S., Cuntz U. (2006). Serial analysis of stable nitrogen and carbon isotopes in hair: Monitoring starvation and recovery phases of patients suffering from anorexia nervosa. Rapid Commun. Mass Spectrom..

